# Effects of taxifolin from enzymatic hydrolysis of *Rhododendron mucrotulatum* on hair growth promotion

**DOI:** 10.3389/fbioe.2022.995238

**Published:** 2022-09-08

**Authors:** Sun-Min Park, Yi-Chang He, Chun Gong, Wei Gao, Young-Soo Bae, Chuanling Si, Kwang-Hyun Park, Sun-Eun Choi

**Affiliations:** ^1^ Department of Forest Biomaterials Engineering, College of Forest and Environmental Sciences, Kangwon National University, Kangwon, South Korea; ^2^ Key Lab of Agricultural Resources and Ecology of Poyang Lake Basin, College of Land Resources and Environment, Jiangxi Agricultural University, Nanchang, Jiangxi, China; ^3^ Jiangxi Academy of Forestry, Nanchang, Jiangxi, China; ^4^ Tianjin Key Laboratory of Pulp and Paper, Tianjin University of Science and Technology, Tianjin, China; ^5^ Department of Emergency Medicine and BioMedical Science Graduate Program (BMSGP), Chonnam National University, Hwasun, South Korea; ^6^ Department of Emergency Medical Rescue, Nambu University, Gwangju, South Korea

**Keywords:** *Rhododendron mucrotulatum*, taxifolin, enzyme hydrolysis, dihydrotestosterone, human follicle dermal papilla cells

## Abstract

Flavonoid aglycones possess biological activities, such as antioxidant and antidiabetic activities compared to glycosides. Taxifolin, a flavonoid aglycones, is detected only in trace amounts in nature and is not easily observed. Therefore, in this study, to investigate the hair tonic and hair loss inhibitors effect of taxifolin, high content of taxifolin aglycone extract was prepared by enzymatic hydrolysis. Taxifolin effectively regulates the apoptosis of dermal papilla cells, which is associated with hair loss, based on its strong antioxidant activities. However, inhibition of dihydrotestosterone (DHT), which is a major cause of male pattern hair loss, was significantly reduced with taxifolin treatment compared with minoxidil, as a positive control. It was also confirmed that a representative factor for promoting hair growth, IGF-1, was significantly increased, and that TGF-β1, a representative biomarker for hair loss, was significantly reduced with taxifolin treatment. These results suggest that taxifolin from enzymatic hydrolysis of RM is a potential treatment for hair loss and a hair growth enhancer.

## Introduction

*Rhododendron mucronulatum* (RM) is a deciduous shrub with a height of approximately 2 m and is distributed in some parts of China and Japan and all over Korea (Chung and Lee, 1991). In Korea, petals are commonly used in food, such as in making flower-patterned griddle cakes. In addition, its roots have been used extensively in oriental traditional medicine as remedies for fever, dysuria, and tonic in Korea and China (Lee et al., 2007; Choi et al., 2011; Si et al., 2011; Si et al., 2013a, 2013b). Flavonoids, which are phenolic compounds (Si et al., 2008a; Si et al., 2008b; Si et al., 2009a; Si et al., 2009c), have been found in the RM flowers, leaves, and stems. The phenolic compounds of RM roots have outstanding antioxidant, anti-inflammatory effects and inhibit allergic inflammation (Lee et al., 2005; Lee et al., 2007; Cho et al., 2008; Choi et al., 2011; Jang et al., 2022).

Taxifolin aglycone has been reported to have strong anti-inflammatory activities in the exudative and proliferative phases of inflammation in albino rats (Gupta et al., 1971). In addition, it has been proven to be effective in diabetic cardiomyopathy by inhibiting oxidative stress to regulate mitochondrial function and inhibit cell apoptosis (Sun et al., 2014; Zheng et al., 2021). Moreover, taxifolin exerts cardioprotective effects against diazinon-induced myocardial injury in rats (Najeb et al., 2022). However, it has been reported that taxifolin aglycone is found in complex preparations such as silymarin (LegalonTM), is presented in very small amounts, and is rarely used alone (Kroll et al., 2007; Weidmann, 2012).

Many researchers use hydrolysis to change the structure of a compound (Xie et al., 2018; Xie et al., 2019; Du et al., 2019; Du et al., 2021a; Lu et al., 2019; Yang et al., 2019; Wang et al., 2021; Wang et al., 2020; Liu et al., 2020a; Xu et al., 2020b; Xu et al., 2020c; Liu et al., 2021a; Liu et al., 2021b; Liu et al., 2022a; Xiong et al., 2021a; Xiong et al., 2021b; Liu et al., 2021e; Liu et al., 2021f; Ma et al., 2021; Zhang et al., 2021). For instance, acid hydrolysis and enzymatic hydrolysis are used to separate sugars (El-Zawawy et al., 2011; Jang et al., 2012; Chen et al., 2016; Dai et al., 2020; Dai et al., 2017; Liu et al., 2017b; An et al., 2020; An et al., 2019; Li et al., 2020; Li et al., 2019; Liu et al., 2020b; Du et al., 2021b; Du et al., 2022; Xu et al., 2021b; Xu et al., 2022; Liu et al., 2022b; Liu et al., 2022c; Liu et al., 2022d; Liu et al., 2022f; Li et al., 2022). Compared to acid hydrolysis, enzymatic hydrolysis is an environmentally friendly method, can be performed under mild reaction conditions, and does not require expensive corrosion protection equipment and neutralization after hydrolysis (Fan et al., 2012; Liu et al., 2017a; Liu et al., 2017b; Chen et al., 2020; Lu et al., 2020; Xu et al., 2020a; Xu et al., 2021a; Liu et al., 2021c, 2021d; Liu et al., 2022e). In a previous study on flavonoid compounds using enzymatic hydrolysis, it was reported that 90% of flavonoid glycosides could be converted into aglycone by hydrolyzing flavonoids and pectic oligosaccharides present in the bark of the bergamot (Mandalari et al., 2006). And the bio-activities could be improved by increasing the total soluble phenolic contents and the flavonoid aglycones content of guava leaves tea by enzymatic hydrolysis (Hu et al., 2017; Wang et al., 2018; Yu et al., 2021; Huang et al., 2021). In a previous study, we conducted a study on the treatment of atopic dermatitis using hirsutenonone which was obtained by enzymatic hydrolysis of oregonin (Jeong et al., 2010). Therefore, based on the success of enzymatic hydrolysis in previous studies, in this study, the effect of taxifolin, prepared by enzymatic hydrolysis, on hair loss, which is an intractable skin disease, was investigated.

Hair loss can be caused by various and complex factors such as aging, excessive stress, and genetic factors (Lei and Chuong, 2016). Various factors, such as insulin-like growth factors (IGF-1), B-cell lymphoma 2 (Bcl-2), Bcl-2-associated X protein (Bax), Poly ADP-ribose polymerase 1 (PARP), and Caspase-3 protein, are involved in cell apoptosis (Muller et al., 1994; Philpott et al., 1994; Zhao et al., 2001). To suppress the death of hair dermal papilla cells, it is necessary to understand how to effectively control these factors. In particular, testosterone, which is a type of androgen, is oxidized by 5α-reductase in the hair dermal papilla cells to produce DHT (Dihydrotestosterone). DHT is known to induce male pattern hair loss by inhibiting hair growth by reducing the growth phase of hair dermal papilla cells (Kaufman, 2002; Dhariwala and Ravikumar, 2019). Hair loss treatment targeting cell apoptosis includes minoxidil, a topical agent, and Propecia, an oral agent. However, these treatments are associated with side effects such as cardiac lesions, irritative dermatitis, and serious deformities (Herman et al., 1996; Nguyen et al., 2003; Hagemann et al., 2005).

Notably, among Asians, especially in Korea, hair loss is emerging as a serious social problem that is leading to stress and depression; hair loss lowers self-esteem and interferes with daily life (Kim et al., 2006; Lee and Lee, 2010). Therefore, it is necessary to develop safer treatments for hair loss using natural ingredients.

In this study, to develop hair tonic and hair loss inhibitors with no side effects, high content of taxifolin extract was prepared by enzymatic hydrolysis. In addition, oxidative stress was induced with H_2_O_2_ to investigate the oxidative stress effects on hair loss and the apoptosis control effect of the enzymatic hydrolysis of RM on dermal papilla cells. Further, we studied the effects of taxifolin separated and purified from enzymatic hydrolysis on the production of DHT, which is the direct cause of male pattern hair loss, hair growth-promoting factor IGF-1, and hair loss promoting factor TGF-β control effect.

## Materials and methods

### Preparation of enzymatic hydrolysis of RM

*Rhododendron mucronulatum* root (60 kg) from the Seoul Yangnyeongsi Medicine Market, Republic of Korea, was soaked in 60% EtOH at room temperature for 1 week and then filtered. The filtrate was concentrated and freeze-dried (4.964 kg, RM 60E). Then, 10 g of RM 60E was diluted in distilled water (150 ml, 95%, w/w) and Pectinex XXL, Ultra SP-L (Nobozymes Co. Ltd., Bagsvaerd, Denmark) (4 ml each, total 8 ml, 5%, w/w) were added. The mixture was shaken aerobically at 300 rpm for 24 h at 40°C, heated for 5 min at 85°C to inactivate the enzyme, and centrifuged for 10 min at 10,000 rpm and filtered. Then, the filtrate was fractionated with ethyl acetate, and the ethyl acetate layer extract was concentrated and freeze-dried to obtain 5.38 g.

### Isolation of taxifolin by the enzymatic hydrolysis of RM

**FIGURE 1:**
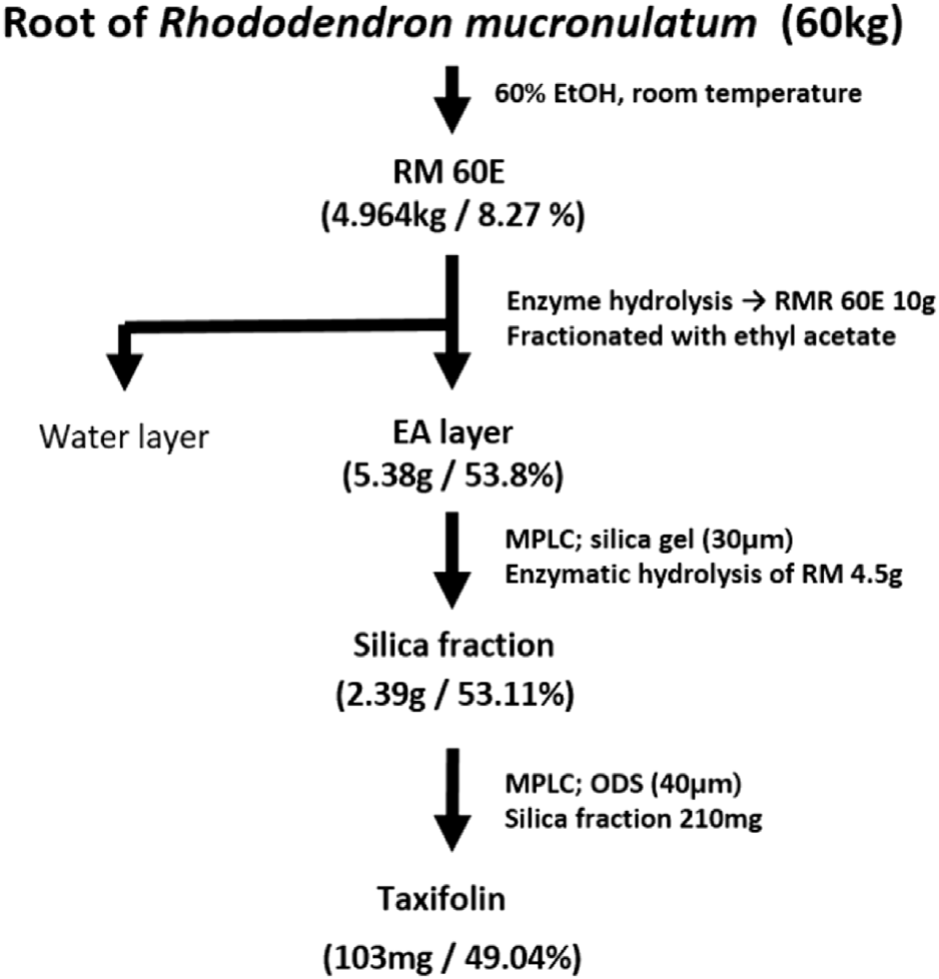
Flowchart of taxifolin isolation process.

To isolate taxifolin (aglycone), Yamazen AI-580S system Medium Pressure Liquid Chromatography (Yamazen Corp., Osaka, Japan) was used. The ethyl acetate layer (4.5 g), and taxifolin (aglycone) were isolated. Silica gel column (30 µm, Yamazen Corp., Osaka, Japan) and ODS column (50 µm, Yamazen Corp., Osaka, Japan) were used. Silica gel column was used with CM solution (chloroform: methanol/5:1) and applied as an isocratic condition. After that, the isolating layer was concentrated and freeze-dried to a powder. Then powder (210 mg) was applied to the ODS column and eluted with distilled water. MW solution (methanol: water) was applied as an gradient condition (30%→35% MeOH; 10 min, 35%→45% MeOH; 20 min, 45%→50% MeOH; 20 min, 50%→0% MeOH; 15 min, 0% MeOH; 5 min). These conditions were repeated to obtain a final 103 mg of single taxifolin. A flowchart of taxifolin isolation is presented in Figure 1.

### Chemical separation and identification

Thin layer chromatography (TLC) was carried out using a pre-coated silica gel 60 F_254_ plate (Merck, Darmstadt, Germany) with chloroform and methanol (5:1, v/v) and chloroform, methanol and water (70:30:4, v/v). The spots were detected under UV radiation (254 nm) and by spraying with ρ -anisaldehyde H_2_SO_4_, FeCl_3_ and 10% H_2_SO_4_ followed by heating. Compound 1 was identified by several instrumental analyses. ^1^H-(700 MHz) and ^13^C-(175 MHz) NMR experiments were recorded with Bruker Avance Ⅲ 700 (Bruker GmbH, Rheinstetten, Germany) at Gyeonggi bio center. Also, Negative LC-MS/MS was conducted with QTRAP 4500 (AB Sciex, Framingham, United States) at Central laboratory Kangwon national university.

### DPPH radical scavenging activity

DPPH radical scavenging assay was measured by modifying the method of Hatano et al. (1989). After preparing the DPPH (Sigma-Aldrich, United States) reagent in 0.1 mM DPPH solution (99.5% methanol, Daejung Chemical & Metals CO., LTD., Gyeonggi, Korea), the reaction ratio between the sample prepared in a varying concentrations in a 96 well plate (NUNC, United States), the sample and DPPH reaction ratio were performed to 1:9 (v/v), The reaction was then performed in the dark for 30 min and absorbance was measured at 517 nm wavelength. The radical scavenging activity was indicated as half-maximal inhibitory concentration (IC_50_) (Re et al., 1999).

### ABTS radical scavenging activity

ABTS radical scavenging assay was measured by modifying the method of Re et al., 1999. ABTS (Sigma-Aldrich, Co., United States) reagent was dissolved in distilled water at a concentration of 7.0 mM, and potassium persulfate (Samchun Chemical Corporation, Korea) was dissolved in H_2_O at a concentration of 2.45 mM. The two solutions were mixed in a 1:1 ratio and left in the dark for 12–16 h to prepare a radical stock solution. The solution was diluted with phosphate-buffered saline, (Welgene, INC., Korea), and absorbance was measured with INNO Microplate Spectrophotometer (LTEK, Seongnam, South Korea) at 750 nm so that an absorbance value between 0.68 and 0.72 was obtained. In a 96 well plate (NUNC, United States), the sample and ABTS reaction ratio were performed to 1:9 (v/v), and the reaction was performed in the dark for 15 min at room temperature. After the reaction, absorbance was measured at 750 nm wavelength. The radical scavenging activity was indicated as half-maximal inhibitory concentration (IC_50_).

### Quantitative chromatographic analysis

High-pressure liquid chromatography (HPLC) was analyzed using a Waters 2,695 system (United States) by modifying the method of Jeon et al., 2011. Experiments were conducted with a combination of 2,487 dual rhamda absorbance detector, run time 40 min, Phenomenex KJ0-4282 Guard column, Sky Pak C18 column (120 Å, 4.6*250 mm, 5 µm), and 25°C of the column oven temperature. A gradient mobile phase consisting of 1% formic acid in distilled water (A) and CH_3_CN (B) was used to run the separation. The elution program was set as follows: from 10% CH_3_CN (B) to 35% CH_3_CN (B) in 20 min, from 35% CH_3_CN (B) to 100% CH_3_CN (B) in 5 min, from 100% CH_3_CN (B) to 10% CH_3_CN (B) in 10 min, and 10% CH_3_CN (B) for 5 min; temperature, room temperature; flow rate, 1.0 ml/min; injection volume, 20 μl; UV detection, 280 nm. Taxifolin (Chengdu Biopurify Phytochemicals Ltd., Chengdu, China) was used for standard. Taxifolin, compound 1, enzymatic hydrolysis of RM and RM 60E were weighed to 1 mg and dissolved in 1 ml of methanol, respectively. Then, taxifolin was diluted to 500, 250, 125, 62.5, and 31.25 ppm and compound 1 was dilluted to 500 ppm.

### Cell culture and treatment

Human follicle dermal papilla cells (HFDPC, # PCS-201-012) and the appropriate media (fibroblast basal medium, #PCS-201-030) and serum-free fibroblast growth kit (#PCS- 201-040 contained human serum albumin, linoleic acid, lecithin), rh FGF β, rh EGF/TGF β-1 supplement, rh insulin and ascorbic acid were obtained from the American Type Culture Collection (ATCC, Manassas, VA, United States). Cells were maintained following by manufacturer’s instructions. The humidified incubator was supplied with 5% CO_2_ maintained at 37°C and different complete media were supplemented with 1% antibiotics and growth factors (ATCC). The cells were used for *in vitro* experiments, after at least 14 days of proliferation.

### MTT assay

Cells were pre-incubated with the indicated doses of taxifolin and treated with H_2_O_2_ for 12 h to investigate the effects of each dose on oxidative-stress-induced cyotoxicity in HDFPC. After incubation, the MTT stock solution (20 μl, final concentration 0.5 mg/ml) of 3-(4,5-dimetylthiazol-2-yl)-2,5-diphenyltetrazolium bromide (MTT, Sigma-Aldrich, MO, United States) was added to each well, and further incubated for 20 min in CO_2_ incubator. After removal of the supernatant, dimethylsulfoxide (100 μl) (DMSO, Sigma-Aldrich, MO, United States) were added to each well. The solubilized formazan after 20 min, the absorbance were measured at an 570 nm using a microplate reader (SPECTROstar Nano, BMG LABTECH, Berlin, Germany).

### Enzyme-linked immunosorbent assay

Enzyme-linked immunosorbent assays (ELISA) were performed for IGF-I (ELH-IGF1-1, RayBiotech, Peachtree Corners, GA, United States), TGF-β (ADI-900-155, Enzo Biochem, New York, NY, United States) and DHT (11-DHTHU-E01, ALPCO, Salem, NH, United States). The sample collection and assay protocols were adapted from the manufacturer’s instructions.

### Statistical analysis

All the experiments were repeated three times, and the mean value and standard deviation were presented. To compare each group, a one-way analysis of variance (ANOVA) followed by Tukey’s multiple range test was used and Student’s *t*-test. SPSS for Windows software (ver. 10.0, Chicago, IL, United States) was used to conduct the statistical analyses. Significance was set at *p* < 0.05.

## Results

### Chemical separation and identification

**FIGURE 2:**
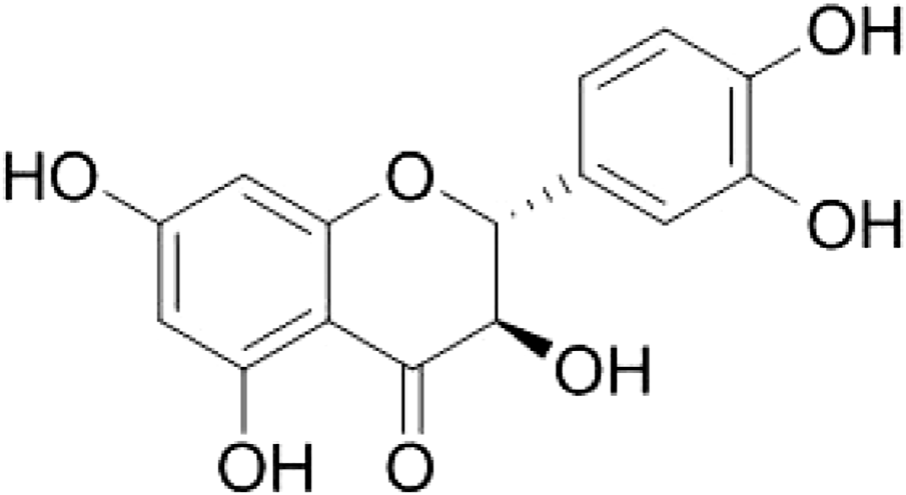
Chemical structures of compound 1. This figure was made with ChemDraw (http://www.perkinelmer.com/category/chemdraw).

Taxifolin: White yellow amorphous powder, Negative LC-MS/MS m/z: 303.0 [M-H]^−^; ^1^H-NMR (700 MHz, MeOH-*d*_4_ + D_2_O): *δ* 6.80–6.96 (3H in total, m, H-2′, H-5′, and H-6′), 5.92 (1H, d, *J* = 2.1 Hz, H-8), 5.88 (1H, d, *J* = 2.1 Hz, H-6), 4.92 (1H, d, *J* = 11.1 Hz, H-2), 4.51 (1H, d, *J* = 11.1 Hz, H-3); ^13^C-NMR (175 MHz, MeOH-*d*_4_ + D_2_O): 198.47 (C-4), 168.74 (C-7), 165.35 (C-5), 164.55 (C-9), 147.18 (C-4′), 146.35 (C-3′), 120.94 (C-6′), 115.91 (C-5′), 116.11 (C-2′), 101.87 (C-10), 97.33 (C-6), 96.30 (C-8), 85.16 (C-2), 73.71 (C-3). The defined chemical structure of purified taxifolin (Figure 2) was made with ChemDraw (Perkinelmer, MA, United States).

**FIGURE 3:**
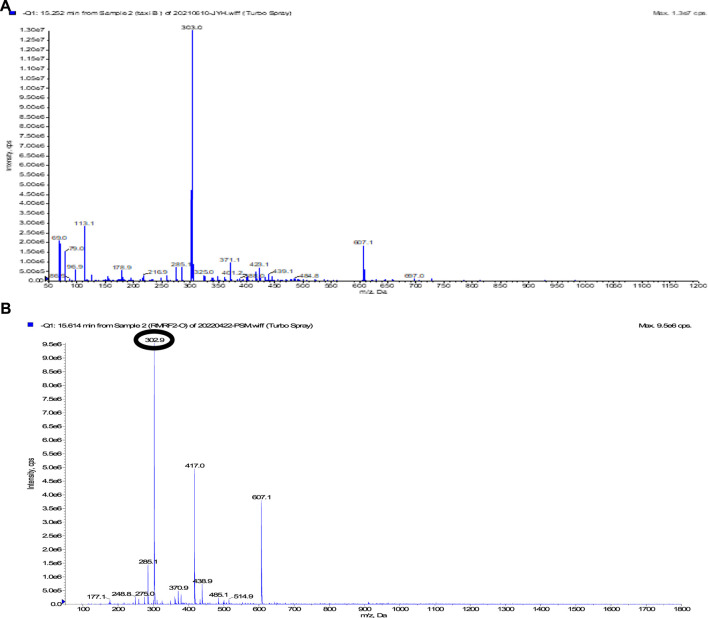
LC-MS/MS spectrum of **(A)** taxifolin and **(B)** compound 1.

Taxifolin was a white yellow amorphous powder. In TLC monitoring, spots were observed under UV radiation at 254 nm. Orange and red spots were detected by heating after spraying with 10% H_2_SO_4_ and anisaldehyde-H_2_SO_4_, respectively. And black spot was confirmed by spraying FeCl_3_. In LC-MS/MS, taxifolin showed 303.0 [M-H]^−^ (Figure 3A) and compound 1 showed m/z: 302.9 [M-H]^−^ (Figure 3B). Therefore, we confirmed that taxifolin and compound had same molecular weight. In ^1^H-NMR spectrum, *δ* 6.80–6.96 (3H in total, m, H-2′, 5′, 6′), a peak presumed to have the ABX type 3,4-dihydroxylation pattern of the B ring appeared. The signals of H-8 and H-6, which are meta-coupled through *δ* 5.92 (1H, d, *J* = 2.1 Hz, H-8), and 5.88 (1H, d, *J* = 2.1 Hz, H-6), respectively, were analyzed. Therefore, it was confirmed that the structure of the A-ring was a 5,7-dihydroxylation pattern. In addition, through *δ* 4.92 (1H, d, *J* = 11.1 Hz, H-2) and *δ* 4.51 (1H, d, *J* = 11.1 Hz, H-3), it was flavanonol structure that had no double bond in the C ring (Supplementary Figure S1). In the ^13^C-NMR spectrum, C-2 and C-3 were 85.16 and 73.71 ppm, respectively, indicating that glycosylation shift had not appeared (Supplementary Figure S2). Therefore, it was confirmed as an aglycone form. Thus, compound 1 was identified as taxifolin aglycone based on the spectral data compared with the values reported in the previous studies (Harborne and Mabry, 1982; Ishimaru et al., 1995; Lee et al., 2005).

**FIGURE 4:**
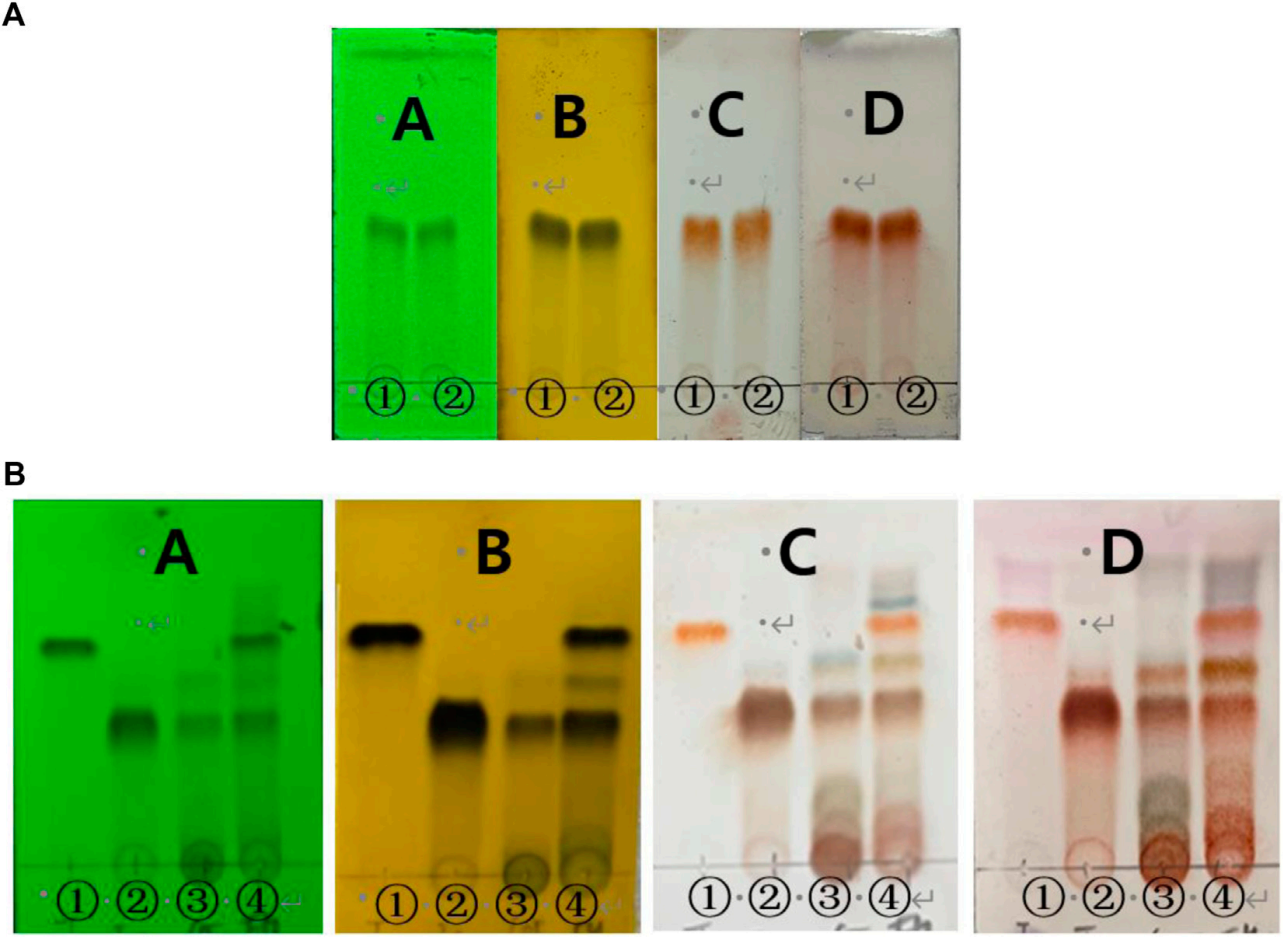
TLC monitoring of **(A)** taxifolin and compound 1. A = UV radiation (254 nm), B = FeCl_3_, C = 10% H_2_SO_4_ D = *ρ*-anisaldehyde H_2_SO_4_. 1 = taxifolin, 2 = compound 1. Chloroform:Methanol = 5:1 and **(B)** compound 1, taxifolin glycoside, RM60E, enzymatic hydrolysis of RM. A = UV radiation (254 nm), B = FeCl_3_, C = 10% H_2_SO_4_ D = *ρ*-anisaldehyde H_2_SO_4_. 1 = compound 1, 2 = taxifolin glycoside, 3 = RM60E, 4 = enzymatic hydrolysis of RM. Chloroform:Methanol:Water = 70:30:4.

In TLC monitoring, compared with taxifolin, spot was shown in compound 1 (Figure 4A). Therefore, we compared taxifolin spot was not confirmed in compound 1, taxifolin glycoside, RM60E and enzymatic hydrolysis of RM (Figure 4B). In enzymatic hydrolysis of RM, taxifolin spot was detected. However, taxifolin spot was not shown in RM60E and only taxifolin glycoside spot was identified.

### DPPH and ABTS radical scavenging activity

**FIGURE 5:**
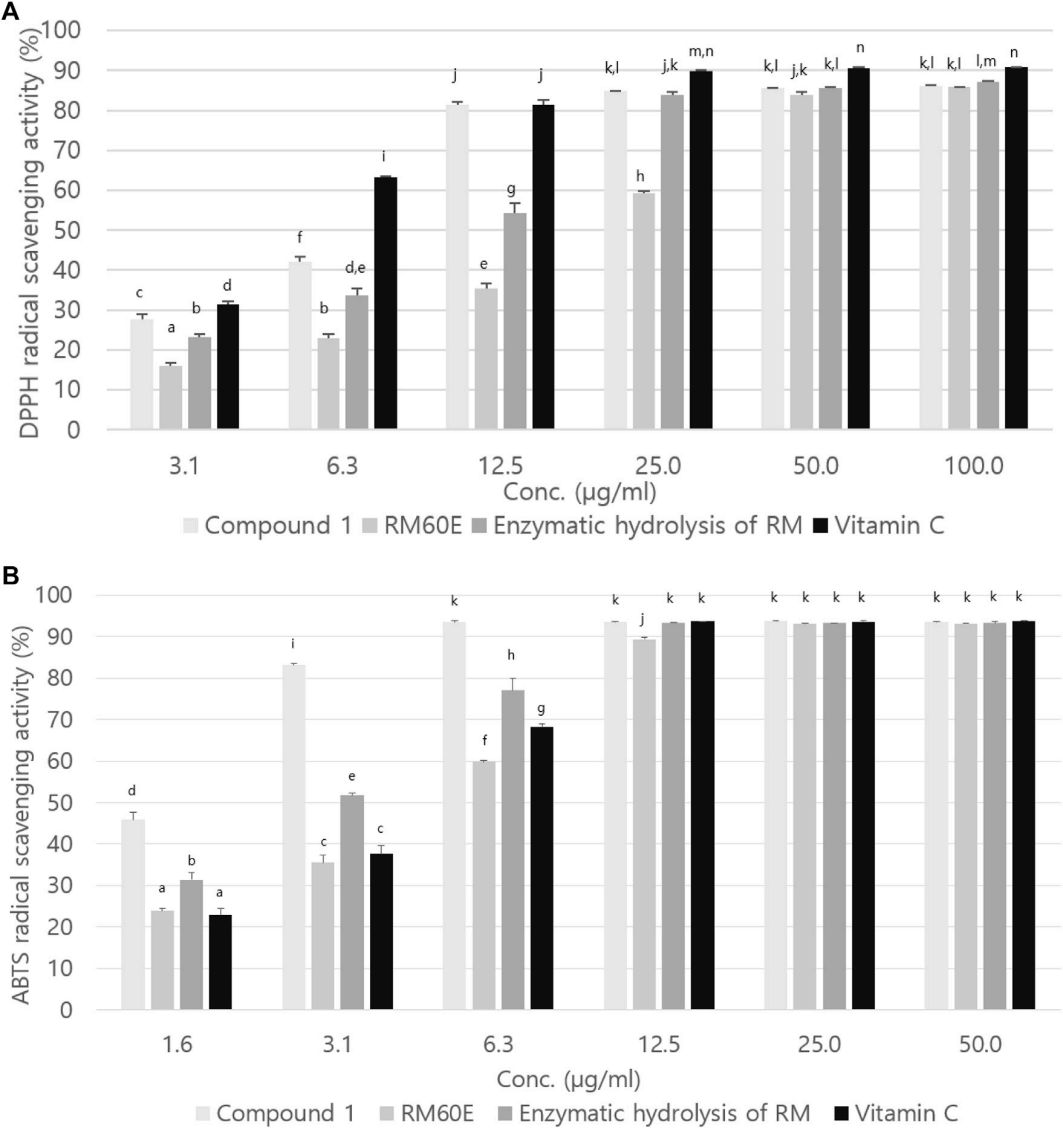
**(A)** DPPH and **(B)** ABTS radical scavenging activities of compound 1, RM60E, and enzymatic hydrolysis of RM. Values were expressed as mean ± S.D. of three determinations. Values bearing different superscripts in the same column are significantly different at *p* < 0.05 by Tukey’s test.

Figure 5A is a graph showing the DPPH radical scavenging activity of compound 1, RM60E and enzymatic hydrolysis of RM. The IC_50_ of compound 1, RM60E and enzymatic hydrolysis of RM was 7.23 ± 0.03 µg/ml, 20.19 ± 0.34 µg/ml, and 11.21 ± 0.73 µg/ml, respectively. The IC_50_ for positive control (vitamin C) was 4.95 ± 0.05 µg/ml.

ABTS radical scavenging activity of compound 1, RM60E and enzymatic hydrolysis of RM is shown in Figure 5B. The IC_50_ of vitamin C, compound 1, RM60E, and enzymatic hydrolysis of RM were 4.36 ± 0.12 µg/ml, 1.68 ± 0.07 µg/ml, 4.98 ± 0.08 µg/ml, and2.99 ± 0.06 µg/ml, respectively.

### Quantitative chromatographic analysis

**FIGURE 6:**
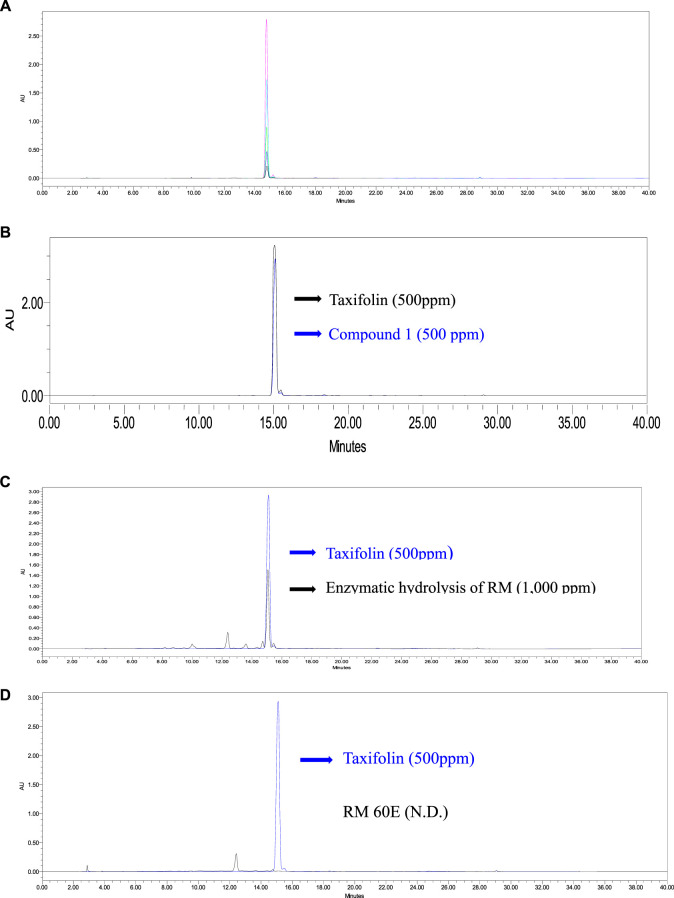
HPLC chromatogram of **(A)** taxifolin. Black (31.25 ppm), blue (62.5 ppm), green (125 ppm), skyblue (250 ppm), pink (500 ppm), **(B)** compound 1 (500 ppm). Black (taxifolin; 500 ppm), blue (compound 1; 500 ppm) **(C)** enzymatic hydrolysis of RM (1,000 ppm). Black (enzymatic hydrolysis of RM; 500 ppm), blue (taxifolin; 500 ppm) and **(D)** RM 60E. Black (RM 60E; 1,000 ppm), blue (taxifolin; 500 ppm).

The relationship between the concentration and the peak-area was measured by the minimum square method (*R* ^2^ value). The standard calibration curve was obtained with concentrations of five increments for taxifolin, *Y* = 79768x − 191089 (*R* ^2^ = 0.9985) as shown in Supplementary Figure S3. Good linearity (correlation coefficient 0.998) is shown for a calibration curve. The retention time of taxifolin was 15.12 ± 0.02 min (Figure 6A). The average content of taxifolin in compound 1 (Figure 6B) and the enzymatic hydrolysis of RM (Figure 6C) and were calculated at 217.82 ± 1.12 µg/ml and 480.27 ± 7.61 µg/ml (*n* = 3) by the above formula. However, taxifolin was not detected in RM 60E (Figure 6D).

### Cell viability of human dermal follicle papilla cell after treatment with taxifolin

**FIGURE 7:**
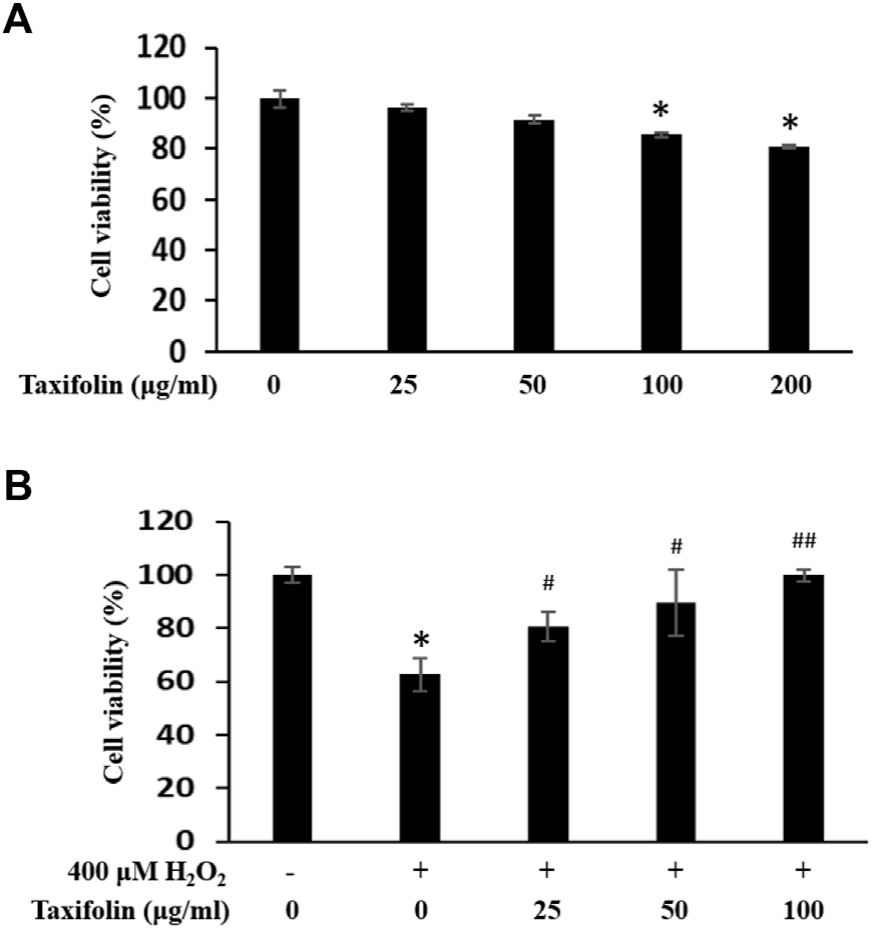
**(A)** Endogenous cytotoxicity of taxifolin in HDFPC. Cells were incubated with the indicated doses of taxifolin. Cell viability was calculated as described in *Materials and methods*. Data are expressed as the Mean ± SD. *p < 0.05 vs. non-treated group. **(B)** Inhibitory effect of taxifolin on hydrogen peroxide-induced cytotoxicity in HDFPC. Cells were pre-incubated with the indicated doses of taxifolin for 20 min and further treated 400 μM H_2_O_2_ for 12 h. Cell viability was calculated as described in *Materials and methods*. Data are expressed as the Mean ± SD. *p < 0.005 vs. non-treated group. ^#^ *p* < 0.05 and ^##^ *p* < 0.005 vs. control group (H_2_O_2_ treated only).

A cytotoxicityeffect of taxifolin was performed on human dermal follicle papilla cells (HDFPC). The HDFPCs were treated with various concentrations of taxifolin, for 12 h. Cell viability was determined by the MTT assay. The endogenous cytotoxicity of taxifolin is presented in Figure 7A. The cell viability of taxifolin (µg/ml) was higher than 80% at nearly 50 µg/ml. The results showed that there was no cytotoxicity observed at all the concentrations.

To investigate the effects of anti-oxidative stress by taxifolin, we examined recovering abilities of taxifolin from enzymatic hydrolysis of RM on H_2_O_2_-induced cytotoxicity model with HDFPC (Figure 7B). Cell viability was markedly decreased to 60% by 400 μM H_2_O_2_ whereas the taxifolin from enzymatic hydrolysis of RM -treated HDFPCs showed a statistically significant increase in antioxidant activities in a dose-dependent manner compared with the control group.

### Efficacy in increasing IGF-1 expression

**FIGURE 8:**
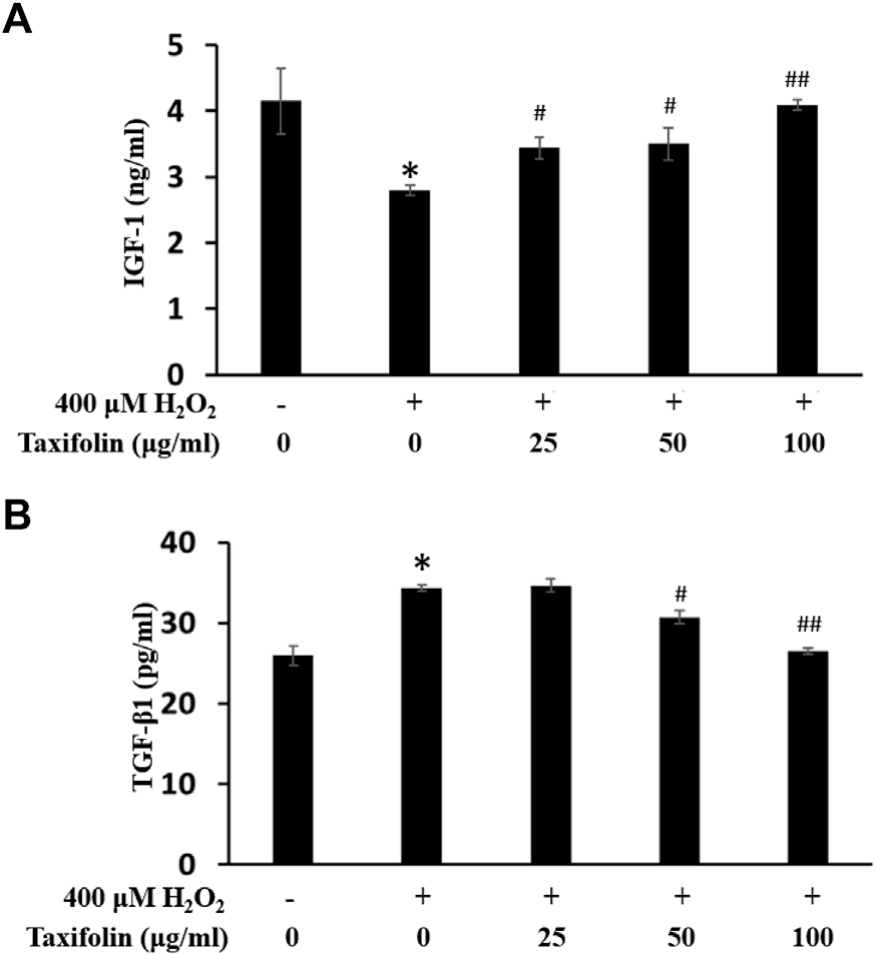
Recovery effects of taxifolin on oxidative stress-induced **(A)** IGF-1 formation and **(B)** TGF-β1 production. Cells were pre-incubated with indicated concentration of taxifolin (μg/mL, w/v) and treated with hydrogen peroxide for 12 h. IGF-1 production as measured as described in *Materials and methods*. Data are expressed as the mean ± SD. *p < 0.001 vs. non-treated group. ^#^ *p* < 0.05 and ^##^ *p* < 0.005 vs. control group (hydrogen peroxide treated only). TGF-β1 production was measured as described in *Materials and methods*. Data are expressed as the mean ± SD. *p < 0.01 vs. non-treated group. ^#^ *p* < 0.05 and ^##^ *p* < 0.01 vs. control group (hydrogen peroxide treated only).

Changes in IGF-1 level on oxidative stress-induced HDFPC damage and recovery effects of taxifolin from enzymatic hydrolysis of RM are shown in Figure 8A. IGF, one of the key maintenance factors involved in the anagen stage of hair growth cycle, was secreted at significantly lower levels by H_2_O_2_ compared normal control group on the intracellular region of HDFPC, whereas pretreatment of taxifolin from enzymatic hydrolysis of RM rescued IGF-1 level in a dose-dependent manner compared to H_2_O_2_ alone. These results suggested that taxifolin from enzymatic hydrolysis of RM was interrupt the oxidative stress-induced malfunction of hair growth cycles.

### TGF-β1 expression inhibition efficacy

As TGF-β families are a well-known inhibitory factors on hair growth and we examined the effect of oxidative stress on the production of TGF-β1. The effect of taxifolin from enzymatic hydrolysis of RM on TGF-β1 expression in the intracellular region was determined (Figure 8B). Treatment with H_2_O_2_ significantly increased TGF-β1 expression compared to the normal controls. However, taxifolin from enzymatic hydrolysis of RM treatment abrogated these effects in a dose-dependent manner, resulting in a significant reduction in TGF-β1 expression compared to H_2_O_2_ treatment alone. These results suggested that taxifolin from enzymatic hydrolysis of RM was regulates the oxidative stress-induced breakdown of hair growth cycles.

### Dihydrotestosterone production inhibitory effect

**FIGURE 9:**
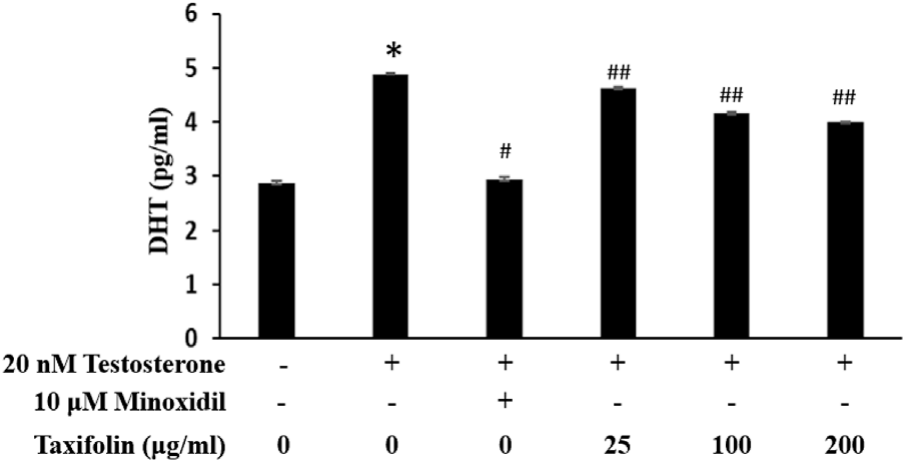
Inhibitory effect of taxifolin on testosterone-induced DHT production in HDFPC. Cells were pre-incubated with the indicated concentration of taxifolin for 20 min and further treated 20 nM testosterone for 12 h. The concentration of DHT were analyzed from cell lysate as described in *Materials and methods*. Data are expressed as the Mean ± SD. *p < 0.001 vs. non-treated group. ^#^ *p* < 0.001 and ^##^ *p* < 0.05 vs. control group (testosterone treated only).

In hair follicle papillae, 5α-reductase, DHT producing enzyme, normally predominated and known androgenic hormone response oi hair follicles depends on an intrafollicular androgenic signaling. The inhibition of DHT was investigated with various concentrations of taxifolin, and 10 µM minoxidil was used in the positive control group (Figure 9). The positive control group treated with minoxidil had a significant and strong inhibitory ability on DHT production. Although a taxifolin from enzymatic hydrolysis of RM was not as potent as minoxidil, it showed a dose-dependent DHT inhibitory effect, and a statistically significant and definite DHT production inhibitory ability when compared with the normal control group.

## Discussion

Taxifolin is known to have various pharmacological properties such as antioxidants, anticancer, cardiovascular, and effective inhibition of oxidative stress, cell apoptosis, malignancies, microbial infection, cardiovascular disease, and liver disease. And it has an anti-cancer activity with a little or no side effects to the normal cells (Si et al., 2009b; Das et al., 2021). In this study, we compared taxifolin with vitamin C, which is positive control. In DPPH radical scavenging activity, especially in low concentration, taxifolin showed significantly high antioxidant activity than RM60E and enzymatic hydrolysis of RM. And in 12.5 µg/ml, taxifolin has antioxidant activity as same as vitamin. Moreover, at a low concentration, the antioxidant activity of taxifolin was higher than that of vitamin C and from 12.5 and above, the results were statistically equivalent to those of vitamin C. Also, it was confirmed that taxifolin has high antioxidants compared with other flavonoids in a previous studies (Lee et al., 2006; Li et al., 2014; Chanput et al., 2016). Therefore, based on the excellent antioxidant activities, we conducted experiments about apoptosis modulation of taxifolin. However, taxifolin is rarely been used alone; it usually used in complex preparations and it is found in small amounts (Weidmann, 2012; Sunil and Xu, 2019). In the previous study, taxifolin barely discernable in the HPLC chromatogram of silymarin (Kroll et al., 2007). According to the quantitative chromatographic analysis, we confirmed that taxifolin was not detected in 60% EtOH extracts from RM. However, high content of taxifolin 217.82 ± 1.12 µg/ml showed in the enzymatic hydrolysis of RM which 217% higher than RM60E. Therefore, it was confirmed that a high content of taxifolin aglycone was produced by enzymatic hydrolysis.

Classically, hair loss is caused by various and complex factors, and one of the causes is oxidative stress. Oxidative stress is known to induce apoptosis then stimulate various cell types within the scalp and hair components; these cell types are keratinocytes, hair follicle cells, papilla cells, and various immune cells. Particularly, apoptosis is mediated by various signal cascades including Bcl-2, Bax, caspase family, and PARP (Tsujimoto et al., 1984; Cleary et al., 1986; Oltval et al., 1993; Muller et al., 1994; Sedlak et al., 1995; Zhao et al., 2001). In our previous reports, when interrupt the oxidative stress can modulate HFDPCs apoptosis (Ha et al., 2021; Kwon et al., 2022). In this study, in H_2_O_2_-induced apoptosis, taxifolin showed statistically significant antioxidant activity. Therefore, it can be expected that taxifolin may modulate the cell death signaling and required further experiments.

IGF-1 is known to be an important growth factor that protects against oxidative stress-induced epithelial cell apoptosis and promotes hair growth (Philpott et al., 1994; Baregamian et al., 2006). Therefore, under the presence of IGF-1, hair follicle morphology was maintained to normal. Furthermore, IGF-1 plays an important role in androgen-dependent alopecia because it is involved in the action of testosterone by affecting the androgen hormone (Price, 1999; Randall, 2008). Thus, we investigated the effect of promoting the expression of growth factors in a dose-dependent manner by treating the dermal papilla cells with taxifolin. The results confirmed that taxifolin will modulate IGF-1 in HDFPC.

TGF-β inhibits the proliferation of hair follicles and it can regulate hair follicles as a negative growth factor. Androgen is related to ROS production, and ROS is involved in TGF-β secretion. Particularly, TGF-β1 secretion is closely related to androgenetic alopecia (Trüeb, 2002) and it focused ROS/antioxidative reaction as crucial role in dermal papilla cells (DPCs) (Shin et al., 2013) as by hair follicle dermal papilla cells (DPCs) in bald scalp. Traditionally, TGF-β was well known as potent inhibitor of the many various cell types (Tucker et al., 1984; Barnard et al., 1988), particularly TGF-β1 was most often mentioned as major factor on hair growth inhibition (Mori et al., 1996; Inui et al., 2003; Shin et al., 2013). Similarly, TGF-β2 (Hibino and Nishiyama, 2004) and predictively TGF-β3 also suggested as important key factor on hair follicle development, growth and cycles (Choi et al., 2018). Moreover, particularly in the epithelial cells with respect to reactive oxygen species (ROS) homeostasis, androgens may be significant in regulating the cellular redox state. In the DP-6 cell line, it is reported that ROS formation was increased after androgen administration, and TGF-β1 secretion was increased by androgen administration (Mori et al., 1996; Park et al., 2001; Olsen et al., 2006). Thus, in hair follicle cells, interruption of the ROS-mediated signaling pathway will inhibit TGF-β1 signals. In this study, we confirmed that taxifolin inhibited TGF-β1 expression in HDFPC. Therefore, taxifolin could be an excellent new natural ingredient for hair growth promotion.

DHT is the representative factor in male pattern hair loss. When testosterone, a male hormone, is oxidized by 5α-reductase, it changes to DHT (Kaufman, 2002; Dhariwala and Ravikumar, 2019). The results of this study showed that with inhibition of DHT production, which is the main cause of male pattern hair loss, with taxifolin, DHT production was modulated in the non-toxic ranges of taxifolin, and, the effects were statistically significant when compared with the positive and negative controls. The positive control group in this experiment, minoxidil, is a common treatment for hair loss. However, minoxidil is only effective for initial hair loss and crown of ongoing hair loss. In the early stages of male pattern baldness, minoxidil can prevent the progression of hair loss. In addition, it was reported that if minoxidil is not applied to the scalp twice a day consistently for 6–12 months, it is not effective. Moreover, minoxidil has side effects, including cardiovascular side effects in children who use minoxidil and irritative dermatitis. And diffuse facial, forearm, cheek and neck hypertrichosis, redness, and contact dermatitis have been reported, and hirsutism can be induced in 0.5%–1% of the users (Hagemann et al., 2005; Georgala et al., 2006; Jimenez-Cauhe et al., 2020). Moreover, there is the disadvantage that hair returns to the state before treatment when minoxidil treatment is stopped (do Nascimento et al., 2020). Therefore, it is important to find effective and safe hair loss treatments. In this study, taxifolin was confirmed to be an effective anti-hair loss agent and a new molecule that promotes hair growth in male pattern hair loss.

## Conclusion

In this study, experiments were conducted to confirm that new molecules derived from natural sources can provide an objective basis for scientifically controlling hair loss and have a low risk of side effects. In general, flavonoid aglycone has stronger biological activities, such as antioxidant activity and antidiabetic activitiesthan glycosides. Taxifolin is detected only in trace amounts in nature and is not easily observed (Weidmann, 2012; Sunil and Xu, 2019). Therefore, after preparing high-content taxifolin aglycone extracts from enzymatic hydrolysis of RM, and separating and purifying taxifolin from the extracts, experiments were conducted. In conclusion, it was confirmed that taxifolin effectively regulates the apoptosis of dermal papilla cells, which are closely related to hair loss, based on its strong antioxidant activity. In addition, DHT, a major cause of male pattern hair loss, was statistically significantly reduced in taxifolin compared to minoxidil treatment. It was also confirmed that taxifolin treatment significantly increased the representative factor for promoting hair growth, IGF-1, and significantly reduced TGF-β1, a representative biomarker for promoting hair loss. These results demonstrated taxifolin as a potential treatment for hair loss resulting from the death of the dermal papilla cells and male pattern hair loss.

## Data Availability

All relevant data is contained within the article: The original contributions presented in the study are included in the article/Supplementary Material, further inquiries can be directed to the corresponding authors.

## Supplementary material

The Supplementary Material for this article can be found online at: https://www.frontiersin.org/articles/10.3389/fbioe.2022.995238/full#supplementary-material

Supplementary Figure S1
^1^H-NMR spectrum of compound 1 (700 MHz, MeOH-*d*_4_+D_2_O).

Supplementary Figure S2
^13^C-NMR spectrum of compound 1 (175 MHz, MeOH-*d*_4_+D_2_O).

Supplementary Figure S3 Calibration curve and equation of taxifolin. *Y* = 79768x − 191089 (*R* ^2^ = 0.9985).

## Abbreviations

RM, *Rhododendron mucrotulatum*; RM 60E, 60% EtOH extract from *Rhododendron mucrotulatum*; DHT, dihydrotestosterone; MTT, 3-(4,5-Dimethylthiazol-2-yl)-2,5-Diphenyltetrazolium Bromide; HFDPC, Human follicle dermal papilla cells; ELISA, Enzyme-linked immunosorbent assay; IGF-I, Insulin-like growth factor-I; TGF-β, Transforming growth factor-beta; Bax, Bcl-2-associated X protein; Bcl-2, B-cell lymphoma 2; PARP-1, Poly ADP-ribose polymerase 1.
