# Supplementary material for: Effects of taxifolin from enzymatic hydrolysis of Rhododendron mucrotulatum on hair growth promotion
Source: Front Bioeng Biotechnol. 2022 Sep 8;10:995238. doi: 10.3389/fbioe.2022.995238 (PMC9492874; doi:10.3389/fbioe.2022.995238)
Supplement: Supplementary file 1 [file DataSheet1.docx]

**Supplement Information**

**Effects of taxifolin from enzymatic hydrolysis of *Rhododendron mucrotulatum* on hair growth promotion**

Sun-Min Park^1#^, Yi-Chang He^2,3#^, Chun Gong^3^, Wei Gao^3^, Young-Soo Bae^1,3^, Chuanling Si^4^*, Kwang-Hyun Park^5,6^* and Sun-Eun Choi^1^*

^1^Department of Forest Biomaterials Engineering, College of Forest & Environmental Sciences, Kangwon National University, 24341, Republic of Korea

^2^College of Land Resources and Environment, Jiangxi Agricultural University, Key Lab of Agricultural Resources and Ecology of Poyang Lake Basin, Nanchang 330045, Jiangxi Province, P.R. China

^3^Jiangxi Academy of Forestry, Nanchang 330032, Jiangxi Province, P.R. China

^4^Tianjin Key Laboratory of Pulp and Paper, Tianjin University of Science and Technology, Tianjin 300457, P.R. China

^5^Department of Emergency Medicine and BioMedical Science Graduate Program (BMSGP), Chonnam National University Gwangju, Gwangju 61469, Republic of Korea

^6^Department of Emergency Medical Rescue, Nambu University, Gwangju 62271, Republic of Korea

**Supplement Fig 1.**


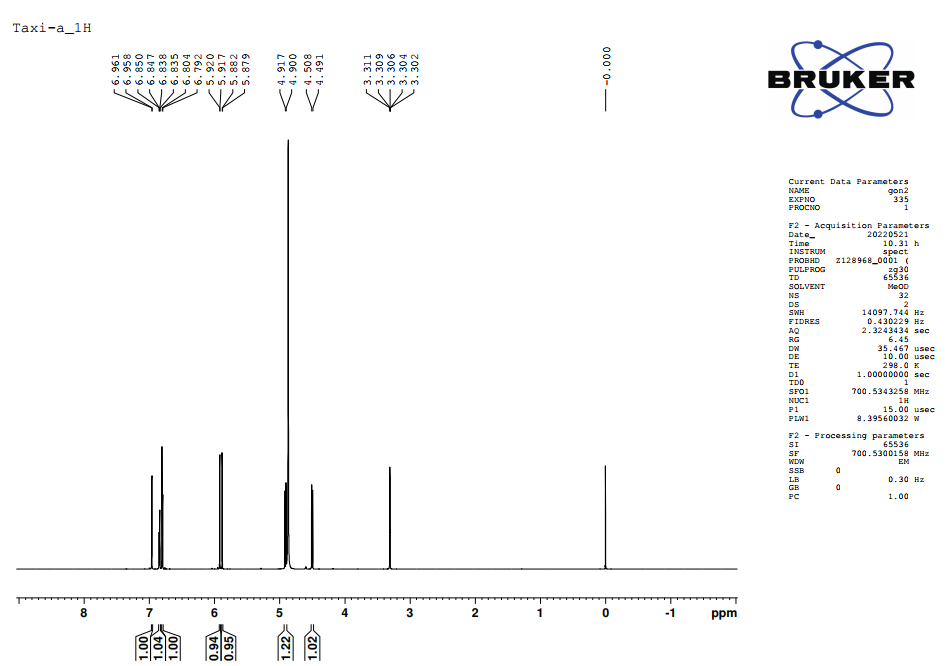


**Supplement Fig. 1**. ^1^H-NMR spectrum of compound 1 (700 MHz, MeOH-*d*_4_+D_2_O).

**Supplement Fig 2.**


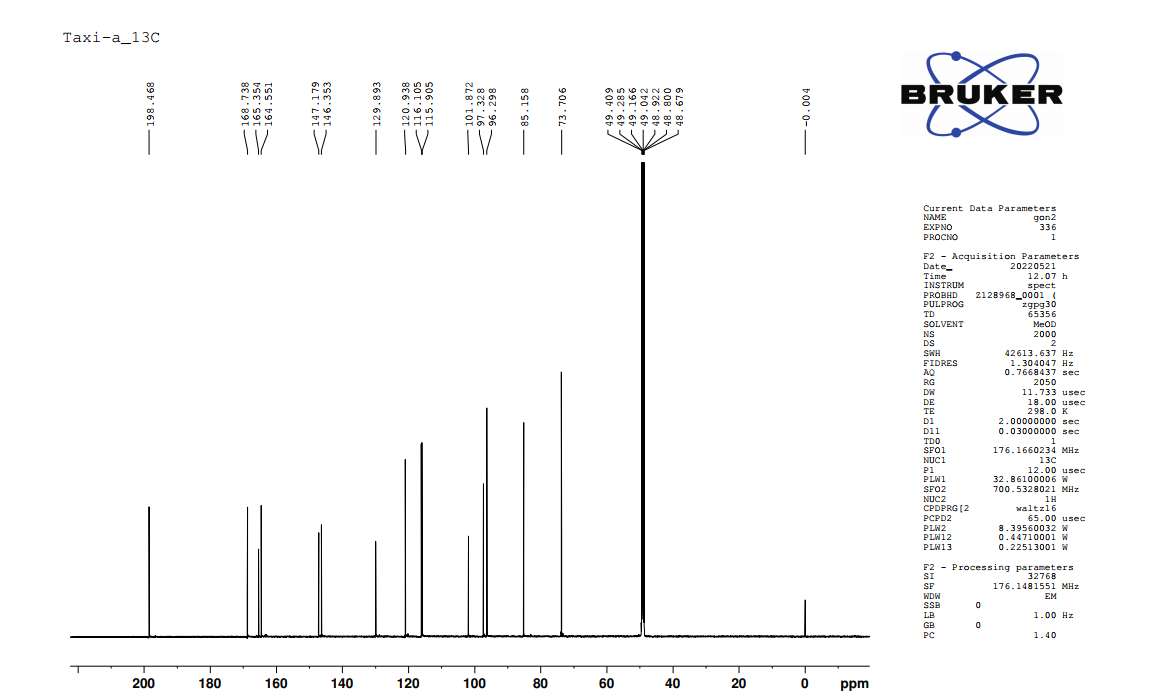


**Supplement Fig. 2**. ^13^C-NMR spectrum of compound 1 (175 MHz, MeOH-*d*_4_+D_2_O).

**Supplement Fig. 3.**

**Supplement Fig.3.** Calibration curve and equation of taxifolin. Y=79768x−191089 (R^2^=0.9985).
